# Supplementary material for: BrewerIX enables allelic expression analysis of imprinted and X-linked genes from bulk and single-cell transcriptomes
Source: Commun Biol. 2022 Feb 17;5:146. doi: 10.1038/s42003-022-03087-4 (PMC8854590; doi:10.1038/s42003-022-03087-4)
Supplement: Supplementary file 1 — Supplementary Information [file 42003_2022_3087_MOESM1_ESM.pdf]

Supplementary information

BrewerIX enables allelic expression analysis of Imprinted and X-linked genes from bulk and single-cell transcriptomes

Paolo Martini[1,2§], Gabriele Sales[1§], Linda Diamante[3], Valentina Perrera[3,4], Chiara Colantuono[5], Sara Riccardo[5], Davide Cacchiarelli[5,6], Chiara Romualdi[1*] and Graziano Martello[1*]

Affiliations:

[1] Department of Biology, University of Padova, Padua, Italy.

[2] Department of Molecular and Translational Medicine, University of Brescia, Brescia, Italy.

[3] Department of Molecular Medicine, Medical School, University of Padova, Padua, Italy.

[4] International School for Advanced Studies (SISSA/ISAS), Trieste, 34136, Italy.

[5] Telethon Institute of Genetics and Medicine (TIGEM), Armenise/Harvard Laboratory of Integrative Genomics, Pozzuoli, Italy

[6] Department of Translational Medicine, University of Naples "Federico II", Naples, Italy

[*] e-mail: chiara.romualdi@unipd.it; graziano.martello@unipd.it

[§] these authors contributed equally to the work

Supplementary Figure 1

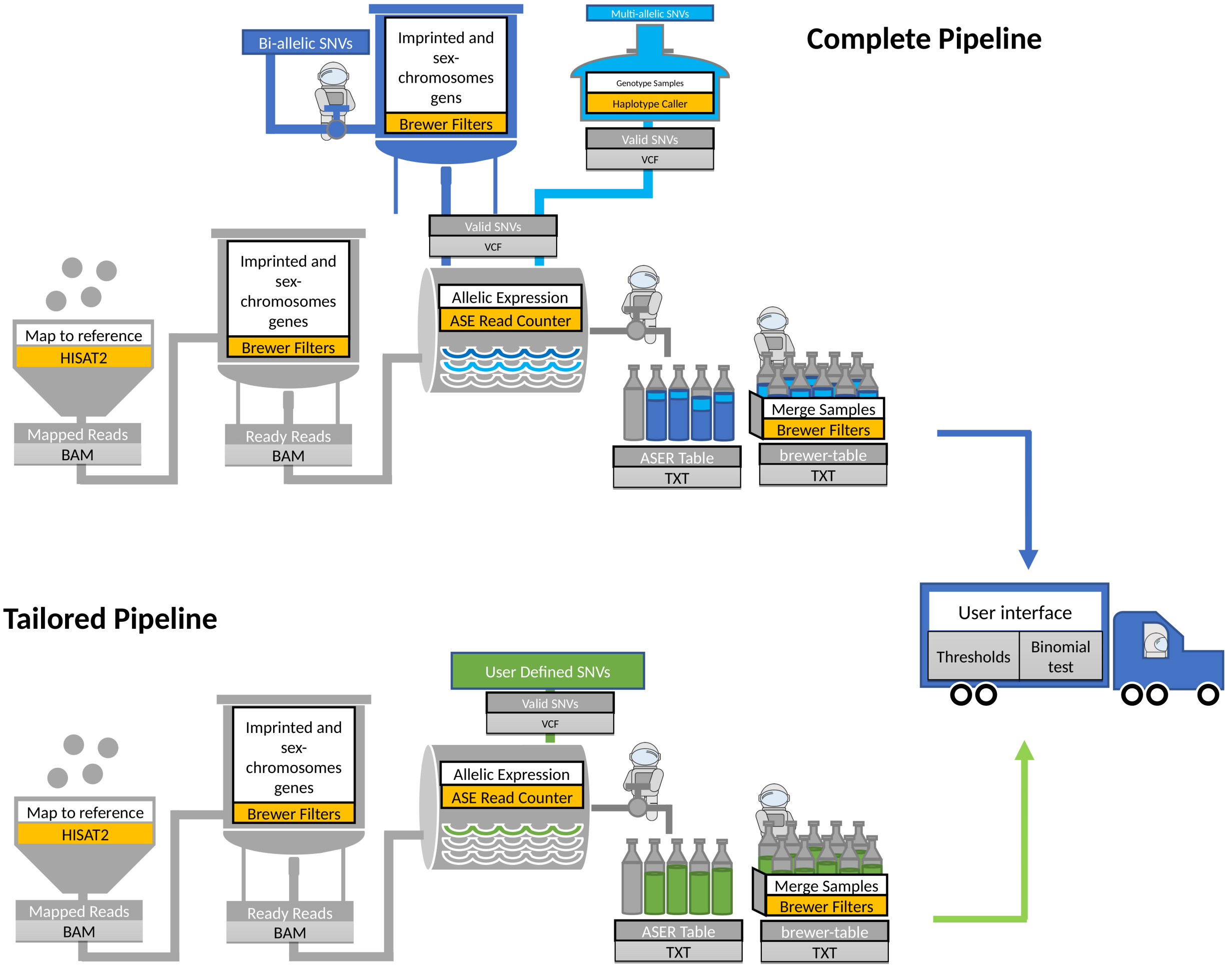

Supplementary Figure 1. Complete and Tailored pipelines overview. The Complete pipeline sacrifices speed for the sake of completeness by using a larger set of SNVs obtained merging i) the SNVs called by HaplotypeCaller (https://gatk.broadinstitute.org) on the user dataset using a pre-compiled set of multi-allelic SNVs and ii) the bi-allelic set used in the Standard pipeline. The use of a larger set of SNVs will increase the power to detect bi-allelic expression. The Tailored Pipeline is meant for users that need to evaluate their own set of SNVs for example those computed using DNA-seq data of matched samples. This allows the user to evaluate imprinting and X-inactivation starting directly from the actual SNV profile of the samples. While the input files for the Standard and the Complete pipelines are only fastq files derived from RNA-seq experiments, the Tailored pipeline additionally requires the VCF file with a set of bi-allelic SNVs. Both Complete and Tailored pipeline results can be further analysed and visualized through the User Interface.

Supplementary Figure 2

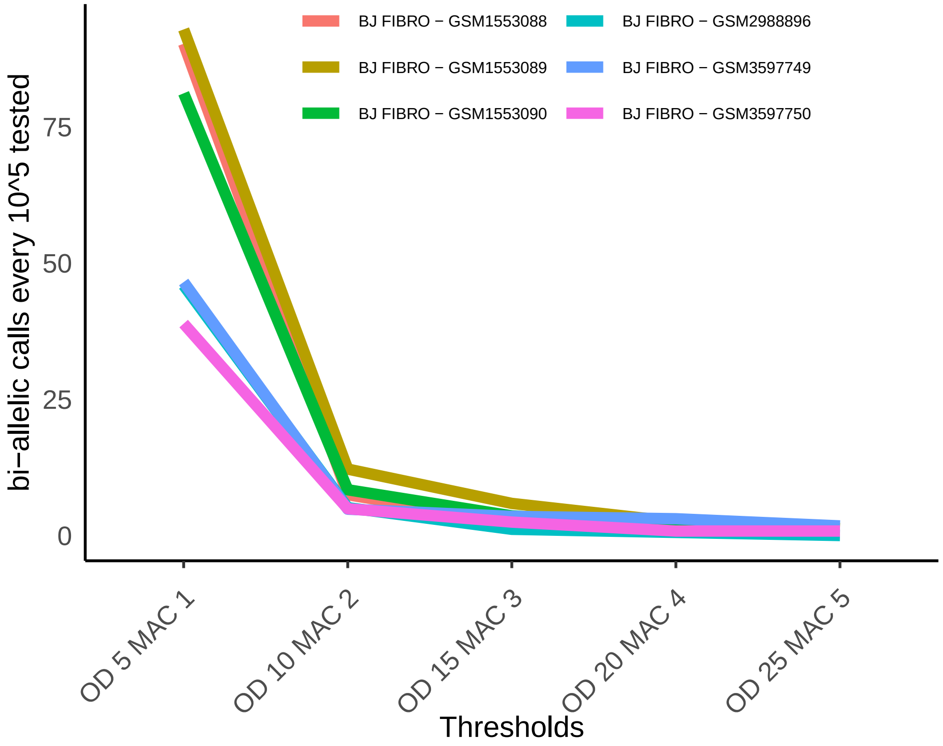

Supplementary Figure 2. False positives bi-allelic calls estimated by analysis of transcripts on the X chromosome in 6 male BJ fibroblasts samples. On the x axis thresholds combination of overall depth (OD) and minor allele coverage (MAC).

Supplementary Figure 3

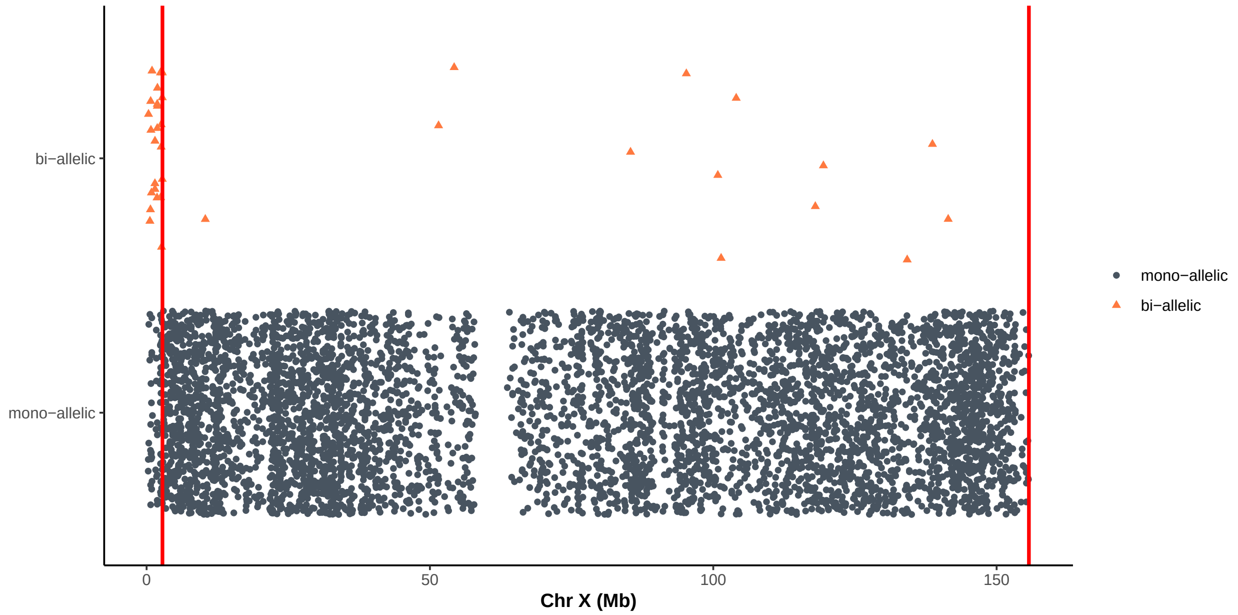

Supplementary Figure 3. BJ fibroblasts genomic DNA analyzed by SNP Array (Affymetrix Mapping 250K Nsp SNP Array, GEO: GSM1868966, series GSE72531). Grey dots are the mono-allelic SNVs. Orange triangles are the bi-allelic SNVs. The vertical red lines indicate the boundaries of PseudoAutosomal Regions (PARs) at both tips of the X chromosome. Excluding the PARs, we observed 13 bi-allelic SNVs out of 5444 analysed (2 every 1,000).

Supplementary Figure 4

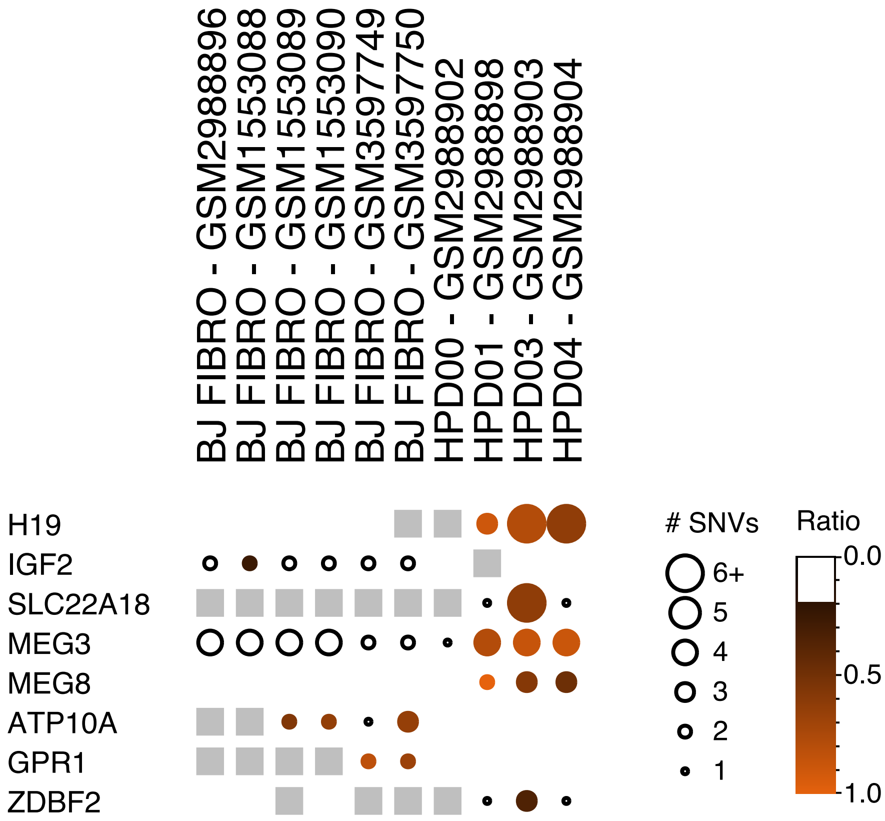

Supplementary Figure 4. BrewerIX gene summary panel. Results on isogenic BJ fibroblasts and both primed and naive iPSCs obtained by BrewerIX using the Standard pipeline. The larger the dot, the higher the number of SNVs supporting the bi-allelic estimate, the darker the orange, the closer to 0.5 is the average of the ratios of all the bi-allelic SNVs. Empty spots are genes with no evidence of bi-allelic expression, white spots are genes with no read overlapping any SNVs

Supplementary Figure 5

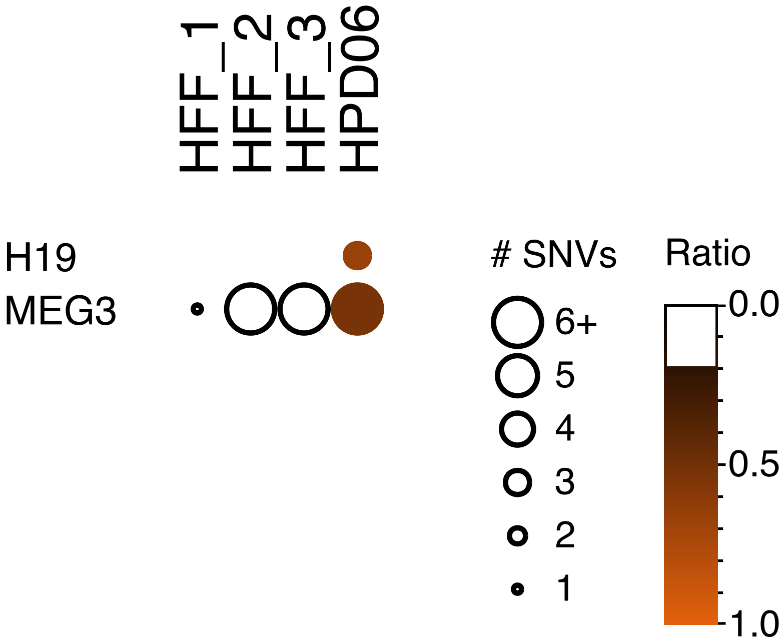

Supplementary Figure 5. BrewerIX gene summary panel. Results on HFF iPS reprogramming dataset (GSE110377) and 3 normal HFF (GSE93226) obtained by BrewerIX in Standard mode. The larger the dot, the higher the number of SNVs supporting the bi-allelic estimate, the darker the orange, the closer to 0.5 is the average of the ratios of all the bi-allelic SNVs. Empty spots are genes with no evidence of bi-allelic expression, white spots are genes with no read overlapping any SNVs.

Supplementary Figure 6

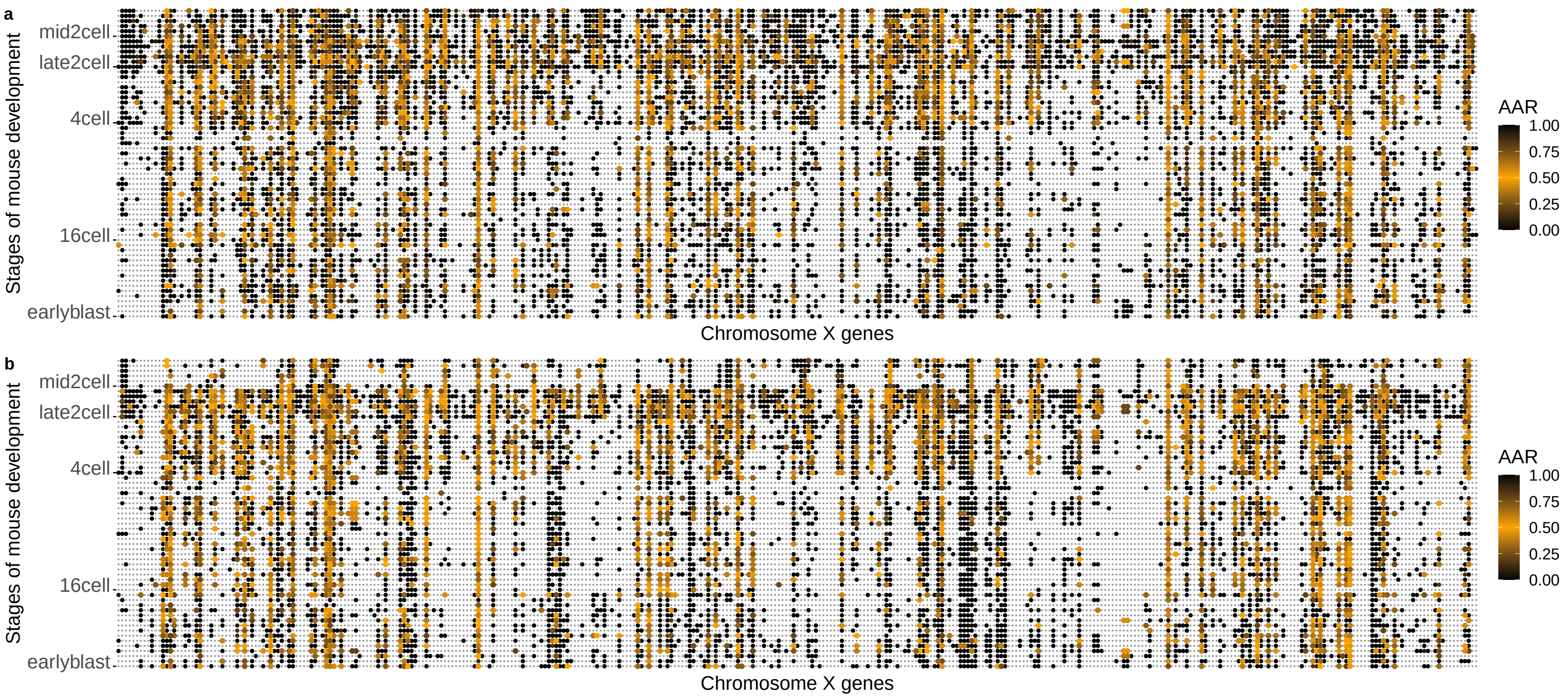

Supplementary Figure 6. X-chromosomes activation status of female samples from dataset GSE45719. Single cells from early mouse embryos at the indicated stage were analysed by single-cell RNA sequencing. a, Average Allelic Ratio of X-chromosome genes (AAR, maternal / total) from the custom analysis performed by Deng and colleagues. b, Average Allelic Ratio of X-chromosome genes (AAR, minor / tot) using BrewerIX computed values. Black dots indicate mono-allelic expression; orange dots indicate bi-allelic expression (AAR=0.5). Grey dots indicate transcripts that were not detected.

Supplementary Figure 7

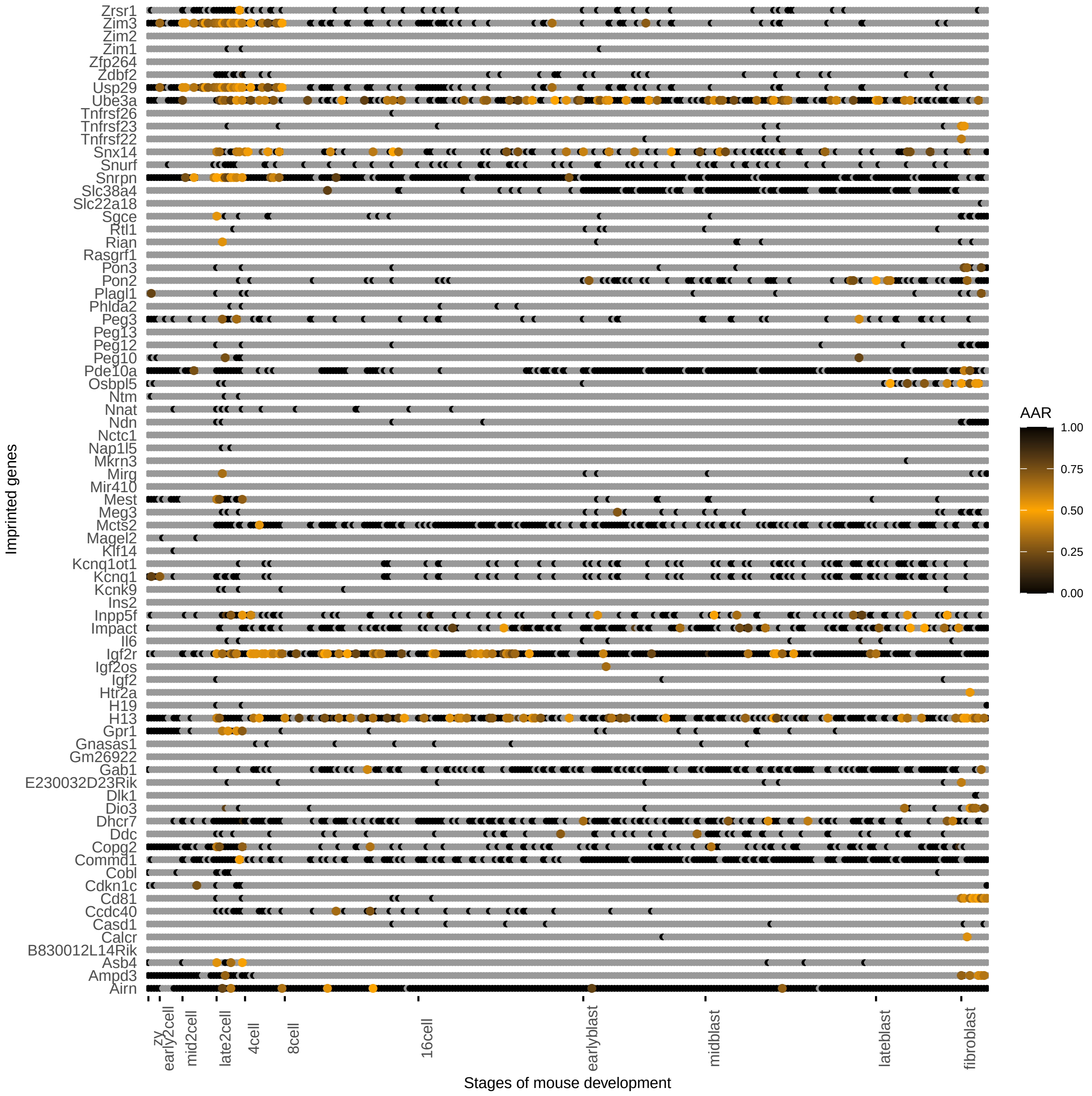

Supplementary Figure 7. Average allelic ratio of imprinted genes across developmental stages computed by BrewerIX on dataset GSE45719. Black dots indicate mono-allelic expression; orange dots indicate bi-allelic expression (AAR=0.5). Grey dots indicate transcripts that were not detected. See also Figure 2c.

Supplementary Figure 8

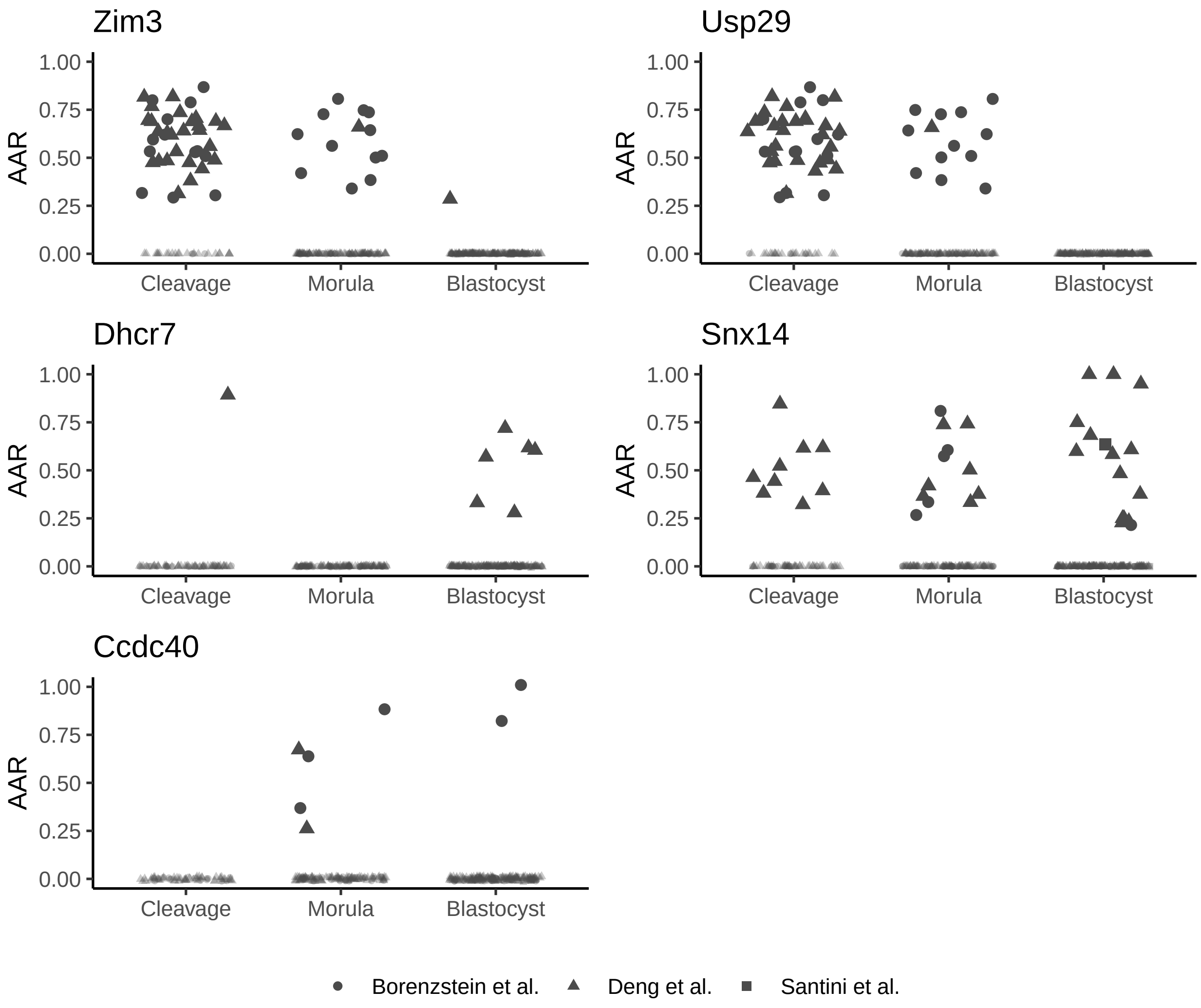

Supplementary Figure 8. Genes with frequent LOI in mouse embrio development obtained by studying three dataset (Borensztein et al., Santini et al., and Deng et al.). On the y axis, the Average Allelic Ratios (AAR) of single samples (single cells or bulk for Santini et al dataset). Developmental stages has been collapsed into broader categories (Cleavage, Morula and Blastocyst).

Description	Datasets	Shown	Species	Type	Samples (n)	Average Depth	Run time Standard mode	Run time Complete mode
Parameter settings - BJ Fibroblasts	GSE110377; GSE126397; GSE63577	Figure 1b, Supplementary Figure 2	Human	Bulk	6	37 M	2.5h	3.5h
Reprogramming of BJ fibroblasts	GSE110377; GSE126397; GSE63577	Figure 1c,d, Supplementary Figure 4	Human	Bulk	10	28 M	3h	4.5h
Reprogramming of HFF Fibroblasts	GSE93226; GSE110377	Supplementary Figure 5	Human	Bulk	3	21 M	1h	1.5h
mESCS - 2i/L and S/L conditions	GSE84164	Figure 1f	Mouse	Bulk	8	22M	2h	3h
mESCs - bulk and single-cell RNAsea	E-MTAB-2600	Figure 1g, Figure2a	Mouse	SC / Bulk	672/3	7 M [70M - 0.004M] / 52M	16h	-
Early mouse development	GSE45719	Figure 2b,c, Supplementary Figure 6-7-8	Mouse	SC	296	22M [139M - 1.3M]	7h	-
Blastocyst-stage embryos	GSE152106	Figure 2c, Supplementary Figure 8	Mouse	Bulk	8	35M [43.9M - 25.3M]		1h
Early mouse development	GSE80810	Figure 2c, Supplementary Figure 8	Mouse	SC	113	18M [85M - 6M]		5h
Human fibroblasts and lymphoblastoid cells	GSE123028	Figure 2d,e	Human	SC	820	18 M [37M - 0.0004M]	20h	-
Breast cancer- bulk and single-cell RNAseq	GSE75688	Figure 2g	Human	SC / Bulk	515/12	5.8M [11.1M - 3.1M] / 10M [7.8M - 4.6M]	5h (SC only)	2.5h (bulk only)
Fetal neocortex and human cerebral organoids	GSE75140	Figure 3, Supplementary Figure 9	Human	SC	734	1.95M [4.02M - 0.01M]	11h	-
ESC-derived MiniBrains	GSE124174	Figure 3, Supplementary Figure 9	Human	SC	3	20.3M; 22.2M; 18.9M	-	1h
iPSC-derived MiniBrains	GSE86153	Figure 3, Supplementary Figure 9	Human	SC	2	301M; 250M	-	3h
ESC and iPSC derived cortical organoids	E-MTAB-8337	Figure 3, Supplementary Figure 9	Human	SC	2	66.9M; 50.6M	-	2h
iPSC derived cortical organoids	GSE112137	Figure 3, Supplementary Figure 9	Human	Bulk	4	23.5M; 10.1M; 9.5M; 26.0M	-	3.5h
ESC and iPSC derived cortical organoids	E-MTAB-8325	Figure 3, Supplementary Figure 9	Human	Bulk	4	25.9M; 17.7M; 35.0M; 29.0M	-	2h

Supplementary Table 1

Supplementary Table 1. List of datasets used for the case studies. Details on number of samples, organism, figure panels where the dataset results are shown, samples' types (bulks or single-cell RNAseq), average sequencing depth per samples, BrewerIX pipeline used and run times on a Linux Gnome Desktop (12 CPU, 32 Gb Ram PC) are reported.

Validation of SNVs by PCR followed by Sanger sequencing								HPD01 Naive iPSCs - GSM2988898		HPD03 Naive iPSCs - GSM2988903		HPD04 Naive iPSCs - GSM2988904	
**SNV**	**Chr**	**position**	**ref**	**alt**	**Gene symbol**	**Sanger sequencing**	**Expression in naive iPSCs (HPD03)**	**Ref**	**Alt**	**Ref**	**Alt**	**Ref**	**Alt**
rs2228613	19	10154917	G	T	DNMT1	confirmed	bi-allelic	23	7	14	33	17	10
rs61750052	19	10154368	G	A	DNMT1	confirmed	bi-allelic	22	7	26	19	16	13
rs2839703	11	1995432	T	C	H19	confirmed	bi-allelic	101	91	92	19	136	57
rs2839704	11	1995429	T	C	H19	confirmed	bi-allelic	102	91	92	20	132	56
rs3741219	11	1995389	A	G	H19	confirmed	bi-allelic	51	47	51	51	79	52
rs2400941	14	100834230	C	G	MEG3	confirmed	bi-allelic	142	115	457	460	150	166
rs8013873	14	100835753	C	T	MEG3	confirmed	bi-allelic	382	267	1128	886	335	271
rs2289998	11	3088178	C	T	OSBPL5	confirmed	bi-allelic	-	-	3	2	-	-
rs2289999	11	3088173	A	G	OSBPL5	confirmed	bi-allelic	-	-	3	2	-	-
rs935431	11	3088067	G	A	OSBPL5	confirmed	bi-allelic	-	-	4	1	-	-
rs11555134	7	50591496	C	T	GRB10	confirmed	biallelic	2	5	-	-	3	2
rs2192206	15	23686360	G	A	NDN	confirmed	bi-allelic	18	9	18	13	27	15

Supplementary Table 2

Supplementary Table 2. List of SNVs validated by PCR followed by Sanger sequencing. Chromosome position, gene symbol, reference and alternative alleles along with their read counts for each sample are reported.

Primer Name	Sequence (5' -> 3')
DNMT1__rs61750052f	CGGCCTCATCGAGAAGAATA
DNMT1__rs61750052r	GTGATCCTCTGGCCTCAGAC
DNMT1__rs2228613f	TCAGCAAGATTGTGGTGGAG
DNMT1__rs2228613r	CCAAACTGGCCTAAATCCAA
GRB10__f	TGGCCATTTCCTTACCTTTG
GRB10__r	GGGTTGACTGAGGAGCAGAG
H19__f	TTCAAAGCCTCCACGACTCT
H19__r	GTCGGAGCTTCCAGACTAGG
MEG3__rs2400941f	CGGGTGTAGACCTCTGAAGC
MEG3__rs2400941r	TTTGGGGCCTGTATGTGAAT
MEG3__rs8013873f	GCCCTCCTGTGGTCTGAGTA
MEG3__rs8013873r	ACGATCACGAGGGGTCTCT
NDN__f	CCCGAATACGAGTTCTTTTGG
NDN__r	TGAATACTGCACTGTAAATCCTGAA
OSBPL5__f	ACCACATCCTCAAATAGGAG
OSBPL5__r	CCTGCAGAGAGGCCAGTG
NAP1L4 __rs7105693f	GGGAAAAGTGTTTTAGGCTTCCA
NAP1L4 __rs7105693r	ACAACTATAGTTTCGGGAGGCA
DLK1__rs1802710f	CTGCAGGTCTTGTCGATGAA
DLK1__rs1802710r	TGGCTTCTCAGGCAATTTCT
UBE3A__rs8179187f	TTTTGCTGCCTACGTGTTTG
UBE3A__rs8179187r	GTACCCCAAGATTGCTTCCA
PEG3__rs34051133f	GGGAGTGCTCAGGCTACATC
PEG3__rs34051133r	CCTCAGCCAGTGTGGGTATT

Supplementary Table 3

Supplementary Table 3. Primers used for PCR and Sanger sequencing
