# Supplementary material for: BrewerIX enables allelic expression analysis of imprinted and X-linked genes from bulk and single-cell transcriptomes
Source: Commun Biol. 2022 Feb 17;5:146. doi: 10.1038/s42003-022-03087-4 (PMC8854590; doi:10.1038/s42003-022-03087-4)
Supplement: Supplementary file 6 — Description of Additional Supplementary Files [file 42003_2022_3087_MOESM6_ESM.pdf]

Description of Additional Supplementary Files

File name: Supplementary Data 1
Description: Human and mouse manually curated imprinted genes.

File name: Supplementary Data 2
Description: BrewerIX called SNVs for validated mono-allelic or bi-allelic expressed genes.

File name: Supplementary Data 3
Description: Source data underlying the graph and charts presented in the main figures.
